# Anger impacts face memory and face – object memory differently in children and adults

**DOI:** 10.1038/s41598-026-39566-5

**Published:** 2026-03-13

**Authors:** Neslihan Onay Forthomme, Ulrike Rimmele

**Affiliations:** 1 Faculty of Psychology and Educational Sciences, University of Geneva, Geneva, 1227 Switzerland; 2 Faculty of Social and Political Sciences, Institute of Psychology, University of Lausanne, Lausanne, 1015 Switzerland

**Keywords:** Memory, Emotion, Anger, Development, Children, Neuroscience, Psychology, Psychology

## Abstract

Angry facial expressions are quickly detected and trigger emotional and physiological responses. They are especially salient in threatening contexts and can alter attention and emotion recognition, particularly in individuals exposed to trauma or violence. These effects extend to memory, individuals with prior violence exposure showing impaired associative memory for angry faces. However, little is known about how angry faces and their context are encoded and retained in typically developing children and adults. In this study, thirty-eight 9- to 11-year-old children (22 girls) and thirty 18- to 30-year-old adults (15 female participants) learned face - object pairs that contained either neutral or angry faces. Memory was tested for faces, objects, and face - object associations. Adults outperformed children in face and associative memory, but not in object memory. Children remembered angry faces better than neutral ones, whereas adults showed no such difference. Angry faces impaired adults’ associative memory but had no effect on children’s associative memory. In both children and adults, memory for objects per se was not different for objects that had been learned with an angry vs. a neutral face. These findings suggest that angry expressions influence memory differently across age groups and memory modalities. In addition, the reduction in adults’ associative memory due to angry faces cannot be fully explained by attentional bias, as object memory remained unaffected. These findings have potential implications for clinical settings.

## Introduction

Angry faces elicit strong emotional responses and elevate physiological arousal in viewers^1^. They are also detected more efficiently than neutral expressions^2^ and are frequently encountered in contexts involving physical or psychological violence. For instance, consider a scenario in which a visibly angry individual stands with a tennis racket moments before committing an act of aggression, while bystanders silently witness the unfolding situation. What do individuals actually remember about such an emotionally intense experience? The angry face? The tennis racket? Other parts of the event? Do children and adults recall the event in similar ways, or are there developmental differences?

While data on emotional memory across development is still scarce, a substantial body of research in adults has shown that emotion can enhance some aspects of memory although impairing others. In particular, emotion differentially impacts item memory (i.e., memory for individual elements) and associative memory (i.e., memory for the link between elements). Negative emotional stimuli are often better remembered than neutral ones^3–6^, reflecting an enhancement in item memory. However, this benefit is accompanied with a trade-off: although memory for emotional items is improved, memory for their associations with other contextual elements and co-occurring peripheral details is often reduced^7–11^.

Beyond the differential effects of emotion on item and associative memory in adults, developmental studies suggest that these memory processes follow distinct trajectories. Although associative memory continues to develop across childhood and adolescence^12,13^, item memory typically reaches adult-like performance earlier^14^.Yet, the developmental trajectory of item and associative memory for emotional stimuli remains underexplored. To address the critical gap how emotion (in particular anger) affects memory in children and adults, it is essential to investigate both item and associative memory for emotional stimuli across developmental stages, including both children and adults. This approach can clarify how emotion impacts memory and contribute to a more comprehensive understanding of the interplay between emotion, memory, and development. Regarding the selection of emotional stimuli, prior clinical research has repeatedly demonstrated altered processing of angry faces in children and adults exposed to adverse life events. Accordingly, employing angry faces as the primary stimulus material provides a stronger translational link between fundamental research of emotion’s effects on memory in children and adults and its applied clinical relevance.

Previous research has demonstrated that early prior exposure to adverse life events, e.g. domestic violence, can alter the processing of threat-related cues later in life, in particular processing of angry faces. Such experiences shape key attentional mechanisms, including a heightened attentional bias^15,16^ and increased sustained attention to angry faces in children^17^. Similar attentional biases have been observed in adults who experienced adverse events during childhood^18^. Emotion recognition abilities are also affected; for instance, children exposed to domestic violence are more likely to misinterpret neutral expressions as angry^19^, a pattern consistent with findings in adults with a history of childhood maltreatment^20^. Considering that prior exposure to violence modulates the processing of angry faces via changes in attentional allocation and emotion recognition, it is plausible that these effects extend to the encoding and retention of memories related to angry faces.

Lambert et al.^21,22^ assessed memory in children and adolescents with a history of violence exposure vs. peers without prior violence exposure. Participants were aged between 8–19 years and completed a memory task comprising face–object pairs^22^ and face–scene pairs^21^. The faces in these studies displayed neutral, happy or angry expressions and were presented alongside objects or scenes. Children and adolescents with a history of violence exposure demonstrated significantly poorer associative memory for angry face–object/scene pairs compared to their peers without such exposure. Notably, this impairment was specific to object/scenes paired with angry faces, whereas no significant differences in associative memory were observed for neutral or happy faces. Furthermore, this pattern remained consistent across age, suggesting that the detrimental effects of violence exposure on associative memory involving angry faces persist throughout development.

These studies provide novel insights into threat processing on memory across development, yet it is important to critically assess their methodological constraints. In these studies, memory was assessed using an associative recognition paradigm, in which participants were presented with previously encoded face–object or face–scene pairs alongside rearranged pairs and asked to identify the correct associations. This approach may not fully isolate associative memory processes, as retrieval failures could stem from deficits in item recognition (i.e. no recognition of the face or object/scene) rather than impaired associative binding^23^. For instance, a participant might incorrectly reject a previously seen face–object pair due to failure in recalling the object, which would reflect item memory failure rather than a deficit in associative memory.

In contrast, cue-based associative memory paradigms where retrieval is prompted by a specific cue offer a more precise measure of associative memory. The hippocampus, known as a convergence zone for binding associations into coherent memory representations^23,24^, has been shown to be selectively activated during cue-based associative tasks^25,26^. Cue-based paradigms more effectively measure the retrieval of associations rather than isolated items, thereby offering a more precise evaluation of associative memory function.

Despite methodological limitations, the findings of Lambert et al.^21,22^ on threat processing on memory across development offered substantial contributions to the field and illuminated important avenues for future research. First, there remains a striking lack of studies examining memory for emotional threats, in particular anger displayed in angry facial expressions, in typically developing children. As a result, our understanding of how angry faces influence memory in normative development is limited. This gap hinders the accurate interpretation of memory difficulties among youth with prior exposure to violence, emphasizing the need for foundational research in non-clinical populations.

The present study aimed to investigate the effects of anger versus neutral expression on memory for faces, adjacent objects, and face – object associations in typically developing children and young adults. Using a cross-sectional design, we examined age group related differences in both item and associative memory in response to emotionally salient (angry) and neutral stimuli. Participants included children aged between 9 and 11 years and young adults, all of whom completed the same task protocol with identical stimuli. During the encoding phase, participants were presented with randomized face–object pairs, in which faces displayed either angry or neutral expressions. They were instructed to form mental imagery linking each face with its corresponding object. Memory performance was assessed in two phases. First, item memory was tested via an old/new recognition task separately for faces and objects. Second, associative memory was assessed using a cue-based forced-choice paradigm. In each trial, participants were shown a cue (a face or an object) and asked to identify the previously paired item from among several options previously seen at encoding. This bidirectional format enabled the assessment of both face-to-object and object-to-face associative memory. As previously demonstrated by Horner and Burgess (2013)^27^, the retrieval of pairwise associations within an event is contingent, meaning that recalling one element of a pair can depend on the successful retrieval of the other. Therefore, this approach could enhance experimental power by increasing the number of associations tested within each pair.

The primary objectives of the study were to assess: memory for angry versus neutral faces, memory for objects paired with angry versus neutral faces, associative memory performance for neutral/angry face – neutral object pairs, and developmental differences in face, object and associative memory between children and adults.

## Results

### Item memory - faces

#### Corrected recognition (Hit minus false alarm)

**Fig. 1:**
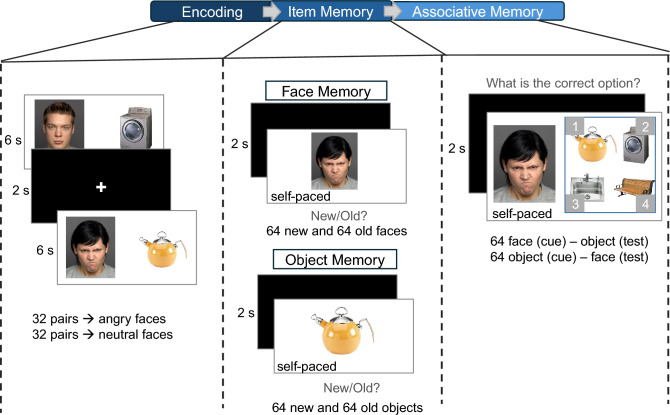
The figure depicts the procedure of the memory task. During the encoding phase, participants were presented with face – object pairings, in which half of the faces displayed neutral expressions and the other half angry expressions. Faces presented in figure are examples of neutral and angry faces from FACES database^57^. Objects were examples from database by Brady et al.^63^. Participants were instructed to generate mental imagery for each trial, integrating the face and object into a coherent scene. Following encoding, participants completed item memory tasks, which consisted of separate face and object recognition tests. In these tasks, previously seen items were intermixed with novel distractors. Participants were asked to indicate whether each image had been presented during encoding by responding “old” or “new.” Subsequently, participants performed an associative memory task. Only items previously encountered during encoding were used as cues or targets. On each trial, either a face or an object appeared on the left side of the screen, accompanied by four response options from the same stimulus category on the right. Participants selected the item originally paired with the cue using the keyboard. The experiment was conducted in a single session without breaks between the encoding and memory phases.

Fixed effects of the fitted mixed model were decomposed with a Type II ANOVA breakdown of 2 (age group: 9- to 11-year-olds and young adults) $\times$ 2 (emotion: neutral and negative) model for corrected recognition analysis. A main effect of age was observed, *F*(1, 61) = 9.13, *p* = .004, $\eta _{p}^{2} = 0.13$, indicating lower corrected recognition of faces in children (*M* = 0.43, *SEM* = 0.02) compared to young adults (*M* = 0.54, *SEM* = 0.03). A main effect of emotion was found, *F*(1, 61) = 13.80, *p* < .001, $\eta _{p}^{2} = 0.18$, showing higher corrected recognition for angry faces (*M* = 0.51, *SEM* = 0.02) compared to neutral faces (*M* = 0.45, *SEM* = 0.02). The interaction of age and emotion was significant, *F*(1, 61) = 7.80, *p* = .007, $\eta _{p}^{2} = 0.11$. Post-hoc analyses indicated that 9- to 11-year-olds’ corrected recognition for angry faces was higher (*M* = 0.49, *SEM* = 0.02) compared to neutral faces (*M* = 0.38, *SEM* = 0.02; *t*[32] = 4.47, *p* < .001, *d* = 0.78). However, the corrected recognition of young adults did not differ between angry faces (*M* = 0.55, *SEM* = 0.03) and neutral faces (*M* = 0.53, *SEM* = 0.04; *p* = .577, *d* = 0.10) (Fig. 1).

#### Hit rates

**Fig. 2:**
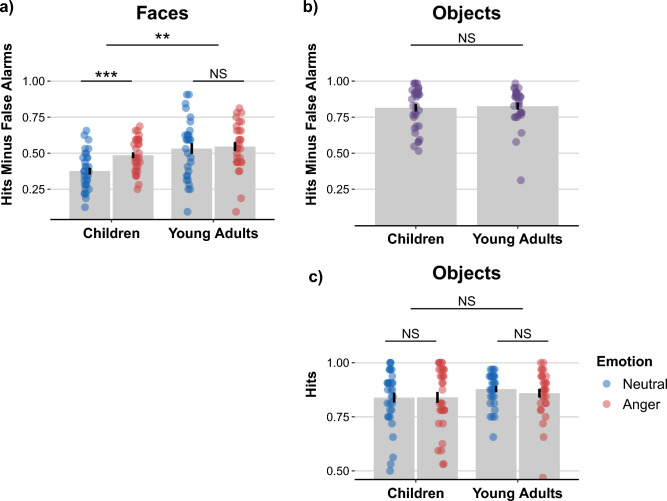
The figure presents the results of the item memory task. In the recognition memory test, children exhibited lower overall face memory performance compared to young adults. Critically, children showed enhanced memory for angry faces relative to neutral faces, whereas young adults’ memory performance did not significantly differ between these emotional expressions (a). Collapsing data across neutral and anger emotion conditions, in the object memory task, children performed comparably to young adults. Corrected recognition scores for objects (i.e., hit rate minus false alarm rate) were similar between the two age groups (b). On the contrary, children and young adults were similarly accurate in correctly identifying previously seen objects as “old” for both objects that had been paired with an angry or with a neutral face during encoding. It was not possible to calculate false alarms (i.e., incorrectly identifying a novel object as previously seen) for each emotion condition, as the new objects presented during the memory task were emotionally neutral (b). Note that for (a) and (c), the left bar represents the neutral emotion condition, whereas the right bar corresponds to the anger emotion condition (a, c ). Data points indicate individual values (a, b, c). Bar plots display mean values, with black lines at the top of each bar representing the standard error of the mean (a, b, c). *p <.05. **p <.01. ***p <.001.

Fixed effects of the fitted mixed model were decomposed with a Type II ANOVA breakdown of 2 (age group: 9- to 11-year-olds and young adults) $\times$ 2 (emotion: neutral and negative) model was conducted for hit rates of faces. This analysis of hit rates for faces mirrored the findings of the corrected recognition scores for faces. A main effect of age group was found, *F*(1, 61) = 18.82, *p* < .001, $\eta _{p}^{2} = 0.24$, demonstrating a lower hit rates for faces in children (*M* = 0.56, *SEM* = 0.02) compared to young adults (*M* = 0.70, *SEM* = 0.03). Emotion main effect was significant, *F*(1, 61) = 6.38, *p* = .014, $\eta _{p}^{2} = 0.09$, with the finding of higher a hit rate for angry faces (*M* = 0.65, *SEM* = 0.02) compared to neutral faces (*M* = 0.61, *SEM* = 0.02). The interaction between emotion and age group was also significant, *F*(1, 61) = 9.20, *p* = .004, $\eta _{p}^{2} = 0.13$. Analyzing children’s data separately, children had a significantly higher hit rate for angry faces (*M* = 0.62, *SEM* = 0.02) compared to neutral faces (*M* = 0.51, *SEM* = 0.02, *t*[32] = 3.65, *p* < .001, *d* = 0.64). Young adults’ hit rates did not significantly differ between angry faces (*M* = 0.69, *SEM* = 0.02) and neutral faces (*M* = 0.71, *SEM* = 0.03; *p* = .623, *d* = 0.09). Results are indicated in Fig. 2.

#### False alarm rates

Fixed effects of the fitted mixed model were decomposed with a Type II ANOVA breakdown of 2 (age group: 9- to 11-year-olds and young adults) $\times$ 2 (emotion: neutral and negative) model was conducted for false alarms of faces. No significant main effect, nor a two-way interaction was found.

### Item memory - objects

Because all objects presented during the encoding phase were neutral, and the novel objects used in the memory task were also neutral, false alarm rates could not be analyzed as a function of emotion condition. Therefore, two-sample t-tests were conducted to examine age group differences for corrected recognition, false alarm rates for objects. However, given that objects had been paired during encoding with angry or neutral faces, we were able to analyze hit rates of the objects depending on emotion condition.

#### Corrected recognition (Hit minus false alarm)

Children (*M* = 0.81, *SEM* = 0.03) did not significantly differ from young adults (*M* = 0.83, *SEM* = 0.02) in corrected recognition scores of objects collapsed across objects that had been paired with angry or neutral faces, *p* = .742, *d* = 0.08. Results are indicated in Fig. 2.

#### Hit rates

Fixed effects of the fitted mixed model were decomposed with a Type II ANOVA breakdown of 2 (age group: 9–11 years, young adults) $\times$ 2 (emotion: neutral, negative) model ANOVA for object hit rates. No significant main effects or interaction were found, i.e. children and adults show similar performance of object memory that had been paired with angry or neutral faces.

#### False alarm rates

When collapsing across emotional conditions, false alarm rates did not significantly differ between children (*M* = 0.02, *SEM* = 0.01) and young adults (*M* = 0.04, *SEM* = 0.01; *p* = .207, *d* = 0.33).

### Associative memory

#### Composite score of associative memory

Fixed effects of the fitted mixed model were decomposed with a Type II ANOVA breakdown of 2 (Age Group: 9- to 11-year-olds vs. young adults) $\times$ 2 (Emotion: neutral vs. negative) model for a composite score of associative memory, i.e. associative memory averaged across the proportion of correctly recalled associations across both testing directions (face [cue] – object [test] and object [cue] – face [test]). A significant main effect of age was observed, *F*(1, 60) = 15.17, *p* < .001, $\eta _{p}^{2}$ = 0.20, indicating that children demonstrated significantly lower composite score associative memory (*M* = 0.60, *SEM* = 0.02) compared to young adults (*M* = 0.75, *SEM* = 0.03). A significant main effect of emotion was also found, *F*(1, 60) = 7.51, *p* = .008, $\eta _{p}^{2}$ = 0.11, with lower memory performance for pairs including an angry face (*M* = 0.66, *SEM* = 0.02) compared to those including a neutral face (*M* = 0.69, *SEM* = 0.02). The interaction between age group and emotion was marginally significant, *F*(1, 60) = 3.67, *p* = .060, $\eta _{p}^{2}$ = 0.06. To further explore this interaction, paired-samples t-tests were conducted within each age group. For children, the composite score of associative memory did not significantly differ between angry face – object pairs (*M* = 0.60, *SEM* = 0.02) and neutral face – object pairs (*M* = 0.61, *SEM* = 0.03; *p* = .561, *d* = 0.10). In contrast, young adults showed a significantly reduced composite score of associative memory for angry face - object pairs (*M* = 0.72, *SEM* = 0.03) compared to neutral face – object pairs (*M* = 0.78, *SEM* = 0.03; *t*[29] = 3.64, *p* = .001, *d* = 0.66).

#### Associative memory for face (cue) - object (test)

Fixed effects of the fitted mixed model were decomposed with a Type II ANOVA breakdown of 2 (Age Group: 9- to 11-year-olds vs. young adults) $\times$ 2 (Emotion: neutral vs. negative) model for associative face – object memory (pairs with faces as presented as a cue and objects as the test elements). Children’s associative memory for face (cue) - object (test) was significantly lower (*M* = 0.68, *SEM* = 0.03) than young adults (*M* = 0.80, *SEM* = 0.03, main effect of age: *F*[1, 60] = 8.36, *p* = .005, $\eta _{p}^{2}$ = 0.12. A main effect of emotion was also observed, *F*(1, 60) = 7.29, *p* = .009, $\eta _{p}^{2}$ = 0.11, showing reduced associative memory for angry face (cue) - object (test) pairs (*M* = 0.72, *SEM* = 0.02) compared to neutral face (cue) – object (test) pairs (*M* = 0.76, *SEM* = 0.02). Interaction between emotion and age group was marginally significant; *F*(1, 60) = 3.24, *p* = .07, $\eta _{p}^{2}$ = 0.05. Post hoc analyses showed that children’s associative memory for angry face (cue) – object (test) pairs (*M* = 0.67, *SEM* = 0.03) were not significantly different from neutral face (cue) – object (test) pairs (*M* = 0.69, *SEM* = 0.03; *p* = .543, *d* = 0.11). On the other hand, young adults’ associative memory for angry face (cue) – object (test) pairs were significantly lower (*M* = 0.77, *SEM* = 0.03) than neutral face (cue) – object (test) pairs (*M* = 0.83, *SEM* = 0.03; *t*[29] = 3.70, *p* < .001, *d* = 0.68).

#### Associative memory for object (cue) - face (test)

**Fig. 3:**
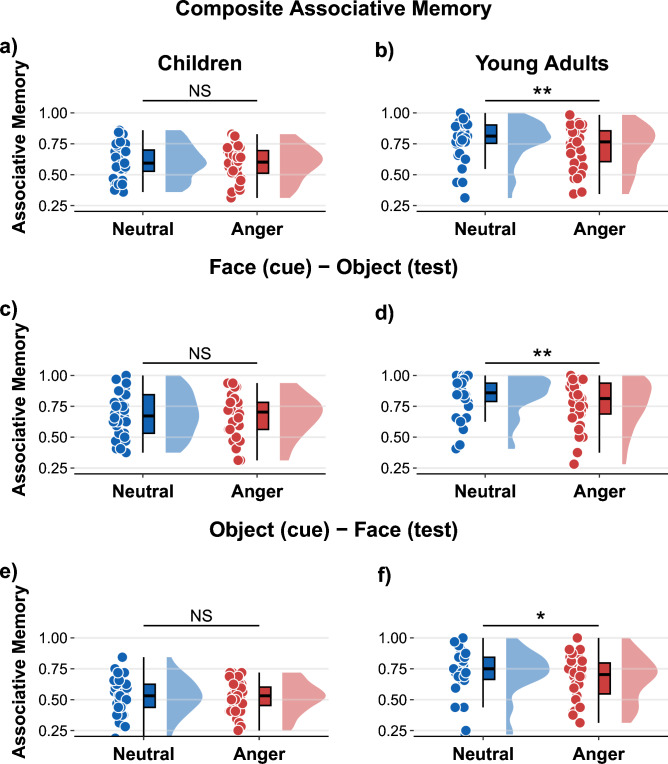
Angry facial expressions differentially influenced associative memory in children and young adults (a, b). Children showed no significant differences in associative memory between neutral face − object pairs and angry face − object pairs. In contrast, young adults exhibited reduced associative memory for angry face − object pairs compared to neutral face − object pairs. Data was aggregated across face (cue) − object (test) and object (cue) and face (test) trials. This reduction in associative memory among young adults occurred regardless of whether the cue was an angry face (d) or an object that had previously been paired with an angry face (f). In children, associative memory performance was similar across neutral and angry conditions (a), both when cued with an angry face (c) and when cued with an object previously presented with an angry face (e). Note that boxplots display individual values as points, along with the distribution of data, for each emotion condition within each age group. *p <.05. **p <.01. ***p <.001.

Fixed effects of the fitted mixed model were decomposed with a Type II ANOVA breakdown of (Age Group: 9- to 11-year-olds vs. young adults) $\times$ 2 (Emotion: neutral vs. negative) model for associative memory of trials objects as presented as a cue and faces as the test elements. Children’s associative memory for object (cue) – face (test) was significantly lower (*M* = 0.53, *SEM* = 0.02) than young adults (*M* = 0.70, *SEM* = 0.03); followed by a main effect of age group, *F*(1, 60) = 21.46, *p* < .001, $\eta _{p}^{2}$ = 0.26. No main effect of emotion (*p* = .102, $\eta _{p}^{2}$ = 0.04) nor an interaction between age group and emotion was found (*p* = .222, $\eta _{p}^{2}$ = 0.02). Results are indicated in Fig. 3.

## Discussion

This study examined how emotional faces, specifically faces expressing anger, affect item and associative memory in children and adults. Participants were tested on their memory for angry and neutral faces, the objects that had been paired with these faces, and the associations between each face - object pair. The results revealed clear developmental differences: adults outperformed children in both face and associative memory, whereas object memory was comparable across age groups. Emotional content influenced memory differently depending on age and memory modality (item: face/object memory vs. associative memory). Children showed enhanced memory for angry faces relative to neutral faces, an effect not observed in adults. In contrast, angry faces impaired associative memory in adults but had no effect on children’s associative memory. In both groups, object memory remained unaffected by the emotional expression of faces with which the objects had been paired . These findings underscore age-group related differences in the effects of anger expression on face and associative memory, whereas revealing similarities for object memory in children and adults.

Regarding age-group effects on item memory (faces and objects per se), our findings of improved memory for faces in adults compared to children, but similar object memory in both age groups align with previous research demonstrating that memory varies between children and adults depending on the category of the item (e.g., faces or objects). The development of category-specific memory has been supported by studies examining both children and adults^28^.In study of Weigelt et al.^28^, researchers found that memory for faces strongly improves with age, whereas memory for other categories (such as objects, bodies and scenes) does not show the same developmental improvement. Along this line, regarding object memory, Ghetti et al.^14^ found a comparable level of object memory among groups of children and adults when tested for their memory for drawings of objects. In contrast to similar object memory, consistent with our findings other researchers also observed that face memory improves childhood to adulthood^29,30^. For example, for recognition memory of faces, Golarai et al.^30^ reported that adults outperformed children aged 7–11 years. This improvement in face memory was linked to increased volume in the right fusiform face area (FFA). In contrast, like in our study, for objects, Golarai et al.^30^observed no age-related differences in object memory. On the neural level, they reported that the volume of the lateral occipital cortex (LOC) in children was comparable to that of adults. Our replication of a category-specific difference in memory (improved face memory, but similar object memory in adults compared to children) together with findings of Golaraia et al.^30^ and previous research^14,28^ suggests that developmental changes in specific brain regions, such as the FFA and LOC, may underlie category-specific developmental improvements in memory. Regarding age effects on associative memory, our findings show that young adults have superior memory performance compared to children for both angry face - object pairs and neutral face - object pairs. This age-group related improvement in associative memory aligns with prior literature. Numerous studies have documented an improvement in associative memory with age using a variety of task protocols for children and adults (e.g., cued recall, associative recognition) and stimulus types (e.g., color, face - object, item - location)^31–35,85^. Previous research has shown that young adults spontaneously employ effective memory strategies, such as mental imagery^36^, whereas children do not favor these effective strategies over less effective ones when memorizing^37^. Instead, children may focus on fantastical and disjointed elements during encoding, which is associated with negative effects on memory^38^. Age-related differences in the use of the encoding strategies required in our experiment, such as different integrative encoding, may also have contributed to differences in associative memory between children and adults.

Examining the influence of emotion on face memory, we found that children recalled angry faces significantly more accurately than neutral ones. In contrast, emotional expression did not affect face memory in adults. Regarding the impact of emotion on item memory in children, our findings align with prior research demonstrating that negative items are retrieved more accurately than neutral items^32,39^. In these studies, both children and adults completed item memory tasks involving images with negative, neutral, and positive emotional valence. The results consistently showed enhanced memory for negative items relative to neutral ones in adulthood. Our findings support the presence of emotion-enhanced item memory in children and adults, although we did not observe a similar effect in adults. The absence of a memory enhancement for angry vs. neutral faces in adults also stands in contrast to previous studies that have reported enhanced memory for negative faces compared to neutral ones in adults^40–44^ and in contrast to studies that report emotion to enhance item memory for different types of stimuli in adults^3,10,11,45^. In these previous studies in adults, participants were presented with faces displaying either neutral or emotional expressions, and memory performance was assessed using recognition tasks^40–44^. Importantly, these studies employed a surprise memory paradigm, whereby participants were unaware that their memory would later be tested and thus they encoded the faces incidentally. The discrepancy between our findings and these studies^40–44^ may be attributable to differences in encoding strategies that adults utilized. In our study, participants were aware of a later memory test, which likely prompted more effortful processing and thus encoding of both angry and neutral facial stimuli. Supporting our results, Bisby et al.^9^ also reported no enhancement in adults’ memory for emotionally valenced images showing individuals similar to those employed in our study. Likewise, Sergerie et al.^46^ found no memory advantage for negative faces over neutral ones in adults. Notably, participants in both studies were aware of the subsequent memory test and completed intentional encoding tasks, mirroring our methodological approach^9,46^. These results suggest that intentional encoding may attenuate the typical emotional enhancement effect observed for item memory in adults^9,46^.

Importantly, developmental differences emerged in the effects of emotion on associative memory, albeit at a marginally significant level. When analyzing each age group separately, we found that angry faces impaired associative memory in adults, whereas children showed no significant difference in performance between angry and neutral face – object pairs. In contrast to our results in children, Lambert et al.^21^ reported enhanced associative recognition for angry face–object pairs compared to neutral ones in children and adolescents, regardless of prior exposure to violence. Notably, they also observed differences in associative recognition memory between participants with and without violence exposure, specifically in trials involving angry face – object pairs. The finding of improved associative recognition for emotional pairs stands in opposition to our results, which showed no significant difference between neutral and angry face – object associations in children. Several methodological differences may account for these discrepancies. In our study, each face was presented only once during encoding, whereas Lambert et al.^21^ manipulated emotional expression while maintaining facial identity in encoding (i.e. presenting the same face with both neutral and angry expressions). This design may have influenced participants’ encoding strategies, making them less comparable to those used in our task. Additionally, the testing procedures differed: Lambert et al.^21^ employed a direct associative recognition task, asking participants to identify previously seen face – object pairings. In contrast, our study used a cue-based associative binding paradigm, targeting the retrieval of the link between face and object. These differences in stimulus presentation and measurement approach may contribute to the divergent findings between our study and that of Lambert and colleagues^21^.

A wide array of studies was conducted with adults testing emotion-related changes in associative memory and consistently demonstrating that emotional items tend to disrupt associative memory relative to neutral ones^7–11,47^. Our findings align with previous research show that adults’ associative memory is fragile in the face of emotional elements. The pattern of impaired associative memory and intact item memory identified in our study was also replicated in another study involving adults^9^. The results may reflect distinct pathways through which emotion enhances item memory and impairs associative memory within medial temporal structures^4,48,49^. Studying object memory, we observed intact performance across both emotional (angry) and neutral conditions, irrespective of age group. Recent evidence suggests that attention may play a critical role in emotional memory impairments^47^. However, several studies indicate that attentional allocation alone does not fully account for these effects. For instance, Rimmele et al.^10^ found no differences in object memory when objects were presented within either neutral or emotional scenes. Supporting our findings, Mickley Steinmetz and Kensinger^50^ observed no increase in eye-tracking fixations for emotional stimuli relative to neutral ones, despite documenting emotion-induced memory impairments. These results imply that post-encoding processes rather than perceptual-level attentional mechanisms may also play a role in mediating the effects of emotion on memory. Thus, although attention may contribute to emotional memory outcomes, it does not appear to fully explain the complex interplay between emotion and memory.

In summary, our study provides novel insights into the influence of angry facial expressions on memory in children and adults, revealing both shared patterns and age-specific effects. Although children demonstrated enhanced memory for angry faces compared to neutral ones, adults exhibited impaired associative memory in the presence of angry faces highlighting a developmental divergence in emotion–memory interactions. The absence of emotion effects on object memory across both age groups further underscores commonalities in item memory. It is also worth noting that the magnitude of difference between the angry and neutral condition was relatively small for some of our measures (e.g. for Composite Score of Associative Memory and Associative Memory in adults). Despite this small difference between means, effect sizes were rather in the range of medium to strong effect sizes suggesting consistent findings^51^. Replication of our research by other researchers and in other populations would enhance robustness of our findings. Previous conceptualizations of post-traumatic stress disorder (PTSD) propose that an imbalance between enhanced memory for negative stimuli and weakened associative memory contributes to intrusive symptoms in adults^48^. In addition, other theoretical frameworks have identified a strong association between altered threat processing following childhood trauma and both externalizing and internalizing forms of psychopathology^52^. By demonstrating empirical evidence on how threatening information, such as angry facial expressions, shapes episodic memory in both children and adults, our findings inform future theoretical models of PTSD as well as conceptual efforts on altered threat learning across a broader spectrum of psychopathologies.

Our study also has some limitations. We did not include within-subject affective measures, which constrains our ability to determine how affective responses relate to memory. Future research should incorporate such metrics, e.g. facial-expression arousal and valence ratings as within-group affect measures, to enhance our understanding of the association between emotion and memory and clarify whether facial-expression arousal and valence ratings contribute to emotion’s impact. Additionally, developmental differences could be more comprehensively examined by incorporating measures such as integrative success, which assesses how well participants are able to perform mental imagery for each trial during encoding (see examples in research of Murray & Kensinger^53^; Bisby et al.^7^). Including such measures would improve our understanding of developmental and emotion variations in memory. Our research also underscores the importance of further research into the cognitive and neural mechanisms underlying emotion effects, particularly in children. Future studies should investigate whether emotional modulation of item and associative memory is governed by distinct processes and examine the extent to which these mechanisms are linked to each other. Furthermore, research should explore how task demands systematically across different age groups (such as incidental versus intentional encoding and immediate versus delayed memory task) influence these emotion–memory interactions. Additionally, examining how stimulus category (e.g., faces vs. objects) shapes these effects would be crucial. A deeper understanding of these mechanisms is essential for developing a comprehensive model of emotional memory development.

## Methods

### Participants

Thirty-eight 9–11-year-old children (22 girls, ${M_{age}}$ = 10.53, ${SD_{age}}$ = 0.70 in years, age range = 9.52–11.90 years) and 30 young adults (15 female participants, ${M_{age}}$ = 23.49, ${SD_{age}}$ = 2.37 in years, age range = 19.45–29.17 years) participated in the experiment. Previous research has shown that there is considerable associative memory development between ages 6 and 8, but between ages 9 and 10 associative memory is stable^13^. Because our primary interest was to examine the within-subjects factor emotion (rather than age), between children and adults, we recruited children aged 9 to 11 to minimize variability within the child age group. We also implemented a two-year age range to facilitate participant recruitment of children. The number of children recruited was slightly higher than the number of young adults as we had expected higher drop-outs or below chance level memory performance in children. Five children were removed from the analysis: two had below-chance level performance in face memory, and three in object memory (ie. <.50). Due to a technical error, one child could not finish the associative memory task; therefore, this child’s data is only included for the face and object memory test. No adult participants were excluded from analysis. The final sample comprised 33 children (19 girls, ${M_{age}}$ = 10.48, ${SD_{age}}$ = 0.67 in years, age range = 9.52–11.64 years) and 30 young adults (15 female participants, ${M_{age}}$ = 23.49, ${SD_{age}}$ = 2.37 in years, age range = 19.45–29.17 years). G*Power^54^ had yielded *N* = 17 per experimental group as the minimum required sample size to achieve a power of 0.80 at an alpha level of .05 (two-tailed), accounting for both within- and between-subjects factor interactions. The experiment employed a 2 $\times$ 2 design, with age group as a between-subjects factor and emotion as a within-subjects factor, analyzed using mixed-design analysis of variance. An expected effect size of 0.25 was specified for Cohen’s $f^2$,corresponding to a medium-to-large effect as defined by Cohen^55^. This estimate was informed by previous studies employing similar experimental paradigms, which reported comparable effect sizes for the influence of emotion^9^ and age group related differences in memory^32,56^.

The experiment with children was conducted in Geneva public schools and the experiment with adults was conducted in the Brain and Behavior Laboratory at the University of Geneva. All methods were performed in accordance with the relevant guidelines and regulations. The study had been approved by the local ethics committee, the Ethics Board of the University of Geneva (CUREG). Children’s parents and the adults provided written informed consent. The children provided their verbal consent to participate in the research. Following the experiment, children received a child magazine, and adults received monetary compensation for their participation.

### Materials

The encoding and the recognition memory tasks included 128 unique faces (64 angry, 64 neutral) and 128 objects. The facial stimuli consisted of child/adolescent faces and adult faces. Adult faces were selected from the FACES database^57^, child and adolescent faces were drawn from the NIMH Child Emotional Faces Picture Set^58^ and the Developmental Emotional Faces Stimulus Set^59^. In order to confirm that an affective reaction is elicited by the faces, we had a separate group of children (*N* = 7, 9–11-years) and young adults (*N* = 10, age: 18–30 years) rate the angry and neutral faces on emotional arousal and valence with the Self-Assessment Manikin (SAM) scale (1 unhappy, 9 happy; 1 calm, 9 = excited^60^). The dimensions of valence and arousal were explained with printed example faces to participants. During the rating task, for each trial, a face appeared at the center of the screen, with the valence and arousal rating scales displayed beneath it. Each trial was self-paced and followed by a 2-second fixation cross. Children’ ratings confirmed, as expected, higher arousal and lower valence for the angry faces (arousal: *M* = 5.96, *SEM* = 0.37; valence: *M* = 2.98, *SEM* = 0.47) than the neutral faces (arousal: *M* = 3.57, *SEM* = 0.40, *t*[6] = 4.65, *p* = .003, *d* = 1.76; valence: *M* = 5.10, *SEM* = 0.23, *t*[6] = −4.85, *p* = .003, *SEM* = 1.83). Likewise, adults’ ratings confirmed higher arousal and lower valence for the angry faces (arousal: *M* = 5.05, *SEM* = 0.61; valence: *M* = 3.88, *SEM* = 0.34) compared to the neutral faces (arousal: *M* = 3.07, *SEM* = 0.36, *t*[9] = 2.94, *p* = .017, *d* = 0.93; valence: *M* = 5.02, *SEM* = 0.07, *t*[9] = −3.45, *p* = .007, *d* = 1.09). We also examined whether there were differences between children’s and adults’ ratings of arousal and valence. The results showed that children’s arousal ratings for angry and neutral faces were comparable to those of adults (*t*[14.02] = 1.27, *p* = .224, *d* = 0.59; *t*[13.66] = 0.92, *p* = .372, *d* = 0.45). Similarly, children’s valence ratings for angry and neutral faces did not differ significantly from those of adults (*t*[11.63] = −1.56, *p* = .147, *d* = 0.78; *t*[7.24] = 0.35, *p* = .735, *d* = 0.18).

Objects were selected based on recent findings about the memorability of objects, which showed that semantic features of an object exert the most significant influence on memorability of an object^61^. Findings from children further show that children’s use of semantic processing is associated with improved recollection of details with age^62^. Therefore, object categories were defined based on previous research and carefully matched across emotion conditions to ensure comparability. This approach minimizes the influence of semantic features on memory performance between the neutral and anger conditions^62^. Objects were selected from the categories defined by Brady et al.^63^, based on their availability. The semantic categories used in our study were determined according to conceptual groupings identified in previous research^61^. Accordingly, the objects in our study were categorized into semantic groups, including clothes (13%), cars/vehicles (6%), and containers (9%), candles and crafts (6%), music instruments (6%), backyard and gardens (6%), electronics (6%), furniture and home décor (6%), hygiene (3%), toys (6%), tools (9%), sport equipment (6%), office supplies (6%). Objects of each of these semantic groups were paired equally with angry and neutral faces.

### Procedure

Each experimental session included the encoding of face - object pairs and three types of memory tests (face memory, object memory, associative memory). To control for potential order effects and participant fatigue, the order of the face and the object memory tests was counterbalanced across participants. In the children’s group, 53% of participants completed the face memory task first, whereas in the young adult group, this was 50%. The remaining participants in both groups completed the object memory task first. The associative memory test was always administered last, as it involved both the faces and the objects from the encoding phase. All memory tasks were self-paced. The total duration of the experiment was about 90 minutes for child participants who completed the full procedure, and about 80 minutes for adult participants. One to six participants were assigned to each experimental session. Procedure is depicted in Fig. 1.

### Memory tasks

#### Practice

The experiment began with verbal instructions. Participants were shown printed images of face - object pairs and were asked to create a story based on the face - object pairs to facilitate encoding. By constructing the mental imagery of a narrative, participants engaged in integrative encoding, whereby the face and object were imagined as components of a unified episodic scene (e.g., a person holding an object), rather than as separate entities. In adults, such integrative encoding has previously been shown to enhance memory compared to non-integrative encoding, i.e. without integration between elements^53^. Following this instruction, participants were presented with individual faces or objects on separate sheets and asked to indicate whether each item was “old” (previously seen) or “new” (not previously seen). Response keys “1” were instructed to use for “old”, i.e. face or object seen during encoding, and “2” for “new” face or “new” object. For the associative memory task, a face was presented on the left side of the paper, along with four objects on the right. Participants were asked to select the object previously paired with the face by replying 1, 2, 3, or 4, corresponding to the correct object. The same example practice trials for face, object, and associative memory tests were also completed individually on a computer and verbal instructions were also presented on the computer screen. Once participants verbally confirmed their understanding of the task instruction and successfully completed the example memory trial on the computer, the experiment started. This procedure indicates that participants were informed that their memory for faces, objects and their association would be assessed, implying that encoding was intentional rather than incidental. Participants were asked if further clarification was needed before the encoding phase began. The main experiment started after confirming that participants understood the task instructions and successfully completed the practice trials on the computer.

#### Encoding

During the encoding phase, participants were shown 64 unique face – object pairs. Half of the faces displayed a neutral expression, and the other half showed an angry expression. Within the angry and neutral face condition, the age and sex of the face were counterbalanced. Therefore, each emotion (neutral vs. angry) condition comprised eight male and eight female child/adolescent faces and eight male and eight female adult faces. Each face was paired with a distinct object. The object categories (e.g., clothes, containers) were balanced across emotion conditions. For example, if two objects from the “clothes” category were shown in the neutral condition, two objects from the “clothes” category were also included in the angry condition. All face – object trials were randomly presented. Each face – object pair was presented for six seconds and followed by a fixation cross for two seconds. The encoding phase was divided into four blocks of 16 trials each. At the end of each block, a prompt appeared instructing participants to press the spacebar to proceed when they felt ready, allowing them to take a short break if needed. Following the encoding phase, participants completed either the face or object memory task first, as determined by the counterbalanced order.

#### Face memory test

During the face memory test, participants were presented with 64 previously encoded faces (32 angry, 32 neutral) alongside 64 novel faces (32 angry, 32 neutral). The new faces were matched to the previously seen faces in terms of sex and age group, following the same counterbalancing strategy as for the set used during encoding. Specifically, the new face set included: eight male and eight female child/adolescent faces and eight male and eight female adult faces for each emotion condition (neutral and angry). The combined set of 128 faces (64 old, 64 new) was presented in a fully randomized order. Participants were instructed to press “1” if they recognized the face as previously seen during the encoding phase (i.e., “old”), and “2” if the face had not been seen in encoding (i.e., “new”). The face memory trials were divided into eight blocks, each containing 16 trials.

#### Object memory test

The object memory task consisted of 64 previously seen (old) objects and 64 novel (new) objects. The new objects were drawn from the same categories used during the encoding phase, and the number of objects per category was matched between old and new sets. For instance, if two objects from the clothes category were presented during encoding, two different objects from the clothes category were included as new objects in the test phase. Thus, participants could not make memory decision based on presentation of objects of a distinct category that had not been presented before.

As for the face memory test, for the object memory test, participants were instructed to indicate whether each object had been seen during encoding or was new. Responses were made by pressing “1” to indicate that the object was “old” (i.e., previously seen) or “2” to indicate that the object was “new” (i.e., not seen during encoding). The object memory test was divided into eight blocks, each consisting of 16 trials.

#### Associative memory test

The associative memory test consisted of 128 trials for which participants completed a cue-based memory task. For 64 trials, faces were used as cues to assess memory for associated objects (face–object pairs). For an additional 64 trials, objects served as cues to assess memory for associated faces (object–face pairs). During each trial, a previously encoded face or object was presented on the left side of the screen, alongside four response options on the right. All response options had been previously shown during encoding.

Participants were asked to select the item (face or object) that was originally paired with the cue during the encoding phase. All faces and all objects were presented once as cue and once as target element. All 128 trials were presented in a randomized order. Each object/face appeared once as a correct (target) response and three times as a distractor across the task. The correct answer (1, 2, 3, or 4) was randomized across trials to minimize response bias. Trials were organized into eight blocks, each containing 16 trials.

### Analysis

#### Face and object memory

To assess face and object memory performance, hit, false alarm, correct rejection, and miss rates were calculated. For face memory, the *hit rate* was defined as the proportion of previously seen (“old”) faces correctly identified as “old” during the memory task. The *false alarm rate* corresponded to the proportion of novel (“new”) faces incorrectly identified as “old.” The *miss rate* represented the proportion of “old” faces incorrectly identified as “new,” and the *correct rejection rate* referred to the proportion of “new” faces correctly identified as “new.” Because hit and miss rates, as well as false alarm and correct rejection rates, are interdependent (i. e. relying on the number of old trials or new trials as denominator), analyses of emotion- and age-related differences were restricted to hit and false alarm rates. Corrected Recognition (defined as hit rate minus false alarm rate) was calculated and submitted to a 2 (age group: 9- to 11-year-olds and young adults) $\times$ 2 (emotion: neutral and angry) analysis of variance to assess the influence of age and emotion on memory for faces. To better understand how hit and false alarm rates contribute to corrected recognition scores, we further analyzed them separately, i.e. we assessed the influence of emotion and age group with a 2 $\times$ 2 ANOVA for separately for hit rates and false alarm rates. Previous research has shown that emotion differentially affects memory for hits and false alarms^64^, and that the developmental trajectories of hits and false alarms across childhood also differ^65^. Accordingly, analyzing hits and false alarms separately allowed us to capture potential distinct effects for each measure.

For object memory, only hit and miss rates were computed specific to emotion condition (had previously been paired with angry or neutral face). All objects presented during encoding were neutral, as were the novel objects presented during the memory task. Therefore, it was not possible to compute false alarm and correct rejection rates for objects specific for the emotion condition. To enable the calculation of *corrected recognition* (defined as hit rate minus false alarm rate) across both emotion conditions, false alarm rates were aggregated across emotion conditions. This corrected recognition score averaged across both emotion conditions was used to assess age-related differences in object memory performance.

#### Associative memory

Associative memory was defined as the proportion of correctly retrieved face (cue) – object (test) or object (cue) – face (test) pairs, calculated separately for each emotion condition (neutral and angry) for each participant. A composite associative memory score was also calculated by averaging associative memory scores across the proportion of correctly recalled associations across both testing directions (face [cue] – object [test] and object [cue] – face [test] to investigate age group and emotion interactions.

### Software information

The experiment was designed and conducted in PsychoPy version 2022.2.2^66^ for memory test and version 2024.2.4 for affect rating. Data processing and data analysis was conducted in R version 4.2.2^67^. For data analysis and statistics, we used, we used *splitstackshape*^68^,*tidyr*^69^, *stringr*^70^, *dplyr*^71^, *writexl*^72^, *rlang*^73^, *readxl*^74^, *purrr*^75^, *plotrix*^76^, *ggpubr*^77^, *lme4*^78^, *lmerTest*^79^, and *stats*^80^, *ggplot2*^81^ packages in R^67^. Data and analysis code are publicly available^82^.

### Statistical analysis

All analyses were conducted using R version 4.2.2^67^. The normality of the data was assessed via Q–Q plots generated using the *ggpubr* package^77^, with visual inspection performed for each level of the interaction.Upon confirming the assumption of normality, the data were analyzed using a linear mixed-effects model approach, implemented via the *lme4*, *lmerTest*, and *stats* packages^78–80^. To account for intra-subject correlation due to repeated measurements, the model specified a random intercept for each participant. Fixed effects were assessed via Type II analysis of variance (ANOVA) employing F-tests, and statistically significant effects were subsequently explored through pairwise comparisons using t-tests. All F-tests were adjusted for the inclusion of the random subject intercept using Satterthwaite’s correction to degrees of freedom. To further explore significant main effects or interactions, post hoc t-tests were conducted. Partial eta-squared values were computed for F-tests as measures of effect size. These values were derived directly from F-statistics using the approximation proposed by Ben-Shachar et al.^83^, which accounts for potential bias due to sample size. For t-test comparisons, Cohen’s d was calculated to quantify effect size. The significance threshold was set at *p* < .05 (two-tailed). Data visualizations were produced using the *ggplot2*^81^ and^ *patchwork*84^ packages. Descriptive data is presented in means and standard error of means.

## Data Availability

Analysis code and data is available in the link : https://osf.io/akhvg.
